# Examining the Relationship between Individualism and Pro-Environmental Behavior: The Moderating Role of Social Cohesion

**DOI:** 10.3390/bs13080661

**Published:** 2023-08-08

**Authors:** Kuk-Kyoung Moon, Seo-Hee Lee, Seo-Yeon Jeong

**Affiliations:** Department of Public Administration, Inha University, Incheon 22212, Republic of Korea; seoh1162@inha.edu (S.-H.L.); 12212871q@inha.edu (S.-Y.J.)

**Keywords:** pro-environmental behavior, individualism, social cohesion, environmental sustainability

## Abstract

Combining Hofstede’s cultural dimensions, value–belief–norm theory, and social exchange theory, this study explores the impact of individualism and social cohesion on pro-environmental behavior (PEB) as well as the moderating role of social cohesion in the individualism–PEB link in the context of Korean society. Using the 2021 Korean General Social Survey and multiple linear regression analyses, we found that individualism is negatively related to PEB, whereas social cohesion is positively related to PEB. Further analysis showed that social cohesion attenuates the negative relationship between individualism and PEB. Our findings suggest that although individuals with high levels of individualism are less likely to perform PEB than those with a high level of collectivism, social cohesion is a valuable community resource that encourages them to engage in eco-friendly activities even when they seek to achieve person-oriented goals and pursue their own interests. The implications and contributions of these findings regarding environmental psychology are discussed.

## 1. Introduction

The natural environment is undergoing rapid deterioration, and the dangers posed by global warming, the loss of biodiversity, air pollution, and other environmental concerns are worse than ever [1,2]. Human activities are widely believed to be the primary cause of these environmental concerns [3]. Thus, there has been considerable scholarly attention in the field of environmental psychology regarding pro-environmental behavior (PEB), which is a set of eco-friendly individual behaviors, including reusing goods, conserving water and electricity, and recycling [1,4]. Indeed, given that PEB contributes to natural environmental sustainability, considerable empirical research has explored the factors that encourage people to engage in PEB and found that demographic characteristics (e.g., gender and age), personal economic situation (e.g., income and home ownership), and environmental attitudes are significant predictors of PEB [1,5].

However, despite considerable evidence of the various antecedents of PEB, research on personal values shaping individual behaviors that are beneficial or harmful to environmental sustainability is scant [1,6]. In this respect, Li et al. [1] recently proposed that researchers should focus on psychological factors to gain insight into how an individual’s desired goals and beliefs lead to their engagement in environmental conservation activities. Individualism is of particular interest in this study because personal values are key drivers of individual behaviors. According to Hofstede’s [7] cultural approach, individuals with high levels of individualism tend to be more concerned with their own self-interests, whereas individuals with collectivism tend to prioritize communal goals. Similarly, the value–belief–norm (VBN) theory suggests that personal values are “psychological antecedents of one’s likelihood to act in a certain manner” ([8], p. 1353), which may shape an individual’s environmentally friendly behavior. Combining the two theoretical perspectives, it is reasonable to predict that individualism oriented toward personal goals is negatively associated with PEB, which contributes to environmental sustainability as a collective interest of the community. In particular, given that personal values and beliefs are powerful predictors of individuals’ behaviors based on VBN theory, individuals who believe that they are independent of other people and have strong self-interest motivation are less likely to engage in PEB because their values and beliefs do not fit with the behaviors that are beneficial to the community environment. By contrast, individuals who prioritize societal benefits and their community’s welfare over their personal interests are more willing to exhibit PEB because their group-oriented goals and norms are consistent with PEB. Based on these theoretical perspectives, our first research question is as follows: “*Are individuals with a high level of individualism less likely to engage in PEB than those with a high level of collectivism?*”

In addition to this difference in personal values, community-related contextual factors play an important role in determining individuals’ behaviors. Social cohesion is an intangible community resource that guides residents to take responsibility for the environment and promotes environmental activities [9]. When the community is cohesive, residents willingly take care of their neighborhoods and help each other [10]. These strong social connections between residents lead to environmental awareness and induce a collective effort to preserve the environment because they perceive that environmental protection enhances the well-being of their community beyond their own interests [11,12]. Consequently, it seems plausible that social cohesion is positively associated with PEB. Furthermore, social cohesion not only directly affects PEB but may also condition the negative relationship between individualism and PEB [10,12]. According to social exchange theory (SET), individuals who perceive interactions with others as valuable and receive desirable treatment from others are likely to reciprocate with positive behavior [13,14]. This social exchange mechanism can be applied to explain the moderating role of social cohesion in the individualism–PEB link [12,15]. In particular, because individualists can see strong emotional connectedness resulting from care and concern received from neighbors and helping behavior shown toward others as favorable gestures, social cohesion encourages them to show behaviors that are beneficial to environmental sustainability as a form of reciprocity of social exchange [16,17]. Accordingly, based on these theoretical perspectives and previous literature, our second and third research questions are as follows: “*Does social cohesion promote PEB?*” and “*How does social cohesion moderate the individualism and PEB relationship?*”

By answering these research questions, our study contributes to the literature on environmental psychology in two ways. First, we advance our understanding of personal values by exploring how individualism is related to PEB. This is particularly important given the scant research attention on how individualism shapes PEB despite the evidence that individuals’ behaviors depend on their values. Our findings may offer clear implications for policymakers and community organizers: individuals who prioritize the “I”-conscious logic of thinking over the “we”-conscious definition of the self ([18], p. 3) are less likely to engage in PEB. Second, this study extends the current knowledge about PEB by examining how social cohesion is associated with PEB and its moderating role in the relationship between individualism and PEB. To this end, we postulate that emotional bonds and solidarity are key resources of the community that encourage PEB and reduce the negative association between individualism and PEB. Moreover, we provide crucial practical implications: policymakers and community organizers should consider implementing various policies to build social cohesion because individuals in a cohesive community are likely to engage in PEB even when they strongly pursue their self-interests.

In the following section, we review the literature on personal values and social cohesion using Hofstede’s cultural framework, VBN theory, and SET to support the study’s hypotheses. We then describe the data and variables employed in the model and explain the findings. We conclude this work by presenting the implications of the results, limitations, and future research directions.

## 2. The Korean Context of the Study

### 2.1. Progressive Transition from Collectivism to Individualism

Long-standing ethnic homogeneity is a significant sociodemographic characteristic of Korea [19]. Consequently, the behavior of individuals is constrained by the implicit shared norms of community members. The emphasis on “cheong” (emotional connection) and “woori” (feeling of “we”) in interpersonal relationships exemplifies the importance of emotional relatedness [20]. Owing to their strong emotional bonds with others in their social communities, Koreans are very in-group-oriented. Indeed, according to Na and Min [21], Koreans commonly develop a strong sense of belonging and a “woori” identity within their in-group. In addition, they often perceive out-groups as separate entities that are distinct from their own [20]. This sense of identification with the in-group is reinforced by emotional bonds and the interconnectedness of extended family networks.

Since the 2000s, collectivist values have gradually shifted toward individualistic values in Korean society, influenced by factors such as advancements in communication technology, economic downturns, and rising costs of childcare and education. For example, in terms of family structure, as of 2021, the vast majority of households in Korea (85.2%) were nuclear families composed of a mother and father and/or their child(ren) [22]; the proportion of couples-only nuclear families also significantly increased, reaching 26.6% compared with 6.4% in 1980 and 14.8% in 2000 ([23], p. 83). Furthermore, single-person households, which accounted for only 4.8% in 1980, reached 15.5% in 2000 and 33.4% in 2021, symbolizing an individualization trend in family composition ([23], p. 82). Accompanying these changes are a delay in marriage and a decline in the marriage rate, which naturally lead to a delay in childbirth and a decline in the birth rate. According to statistics, the percentage of individuals engaging in leisure activities alone reached 51.8% in 2022—significantly higher than the percentage of those engaging in leisure activities with family and friends (33.5% and 12.4%, respectively)—indicating a strong preference for solo leisure activities ([23], p. 41). The advancement of information and communication technology and the development of personalized media consumption have also contributed to the spread of individualism. Kim [24] posited that the shared experience of watching television together is rapidly diminishing, whereas numerous new spaces and personalized forms of entertainment (e.g., YouTube and Instagram) created through individual choices and connections are rapidly expanding. This phenomenon of personal social media accelerated by technological advancements represents the process of individualization in leisure and social networking in South Korea.

### 2.2. An Overview of Korean Pro-Environmental Behavior

Improving social sustainability by decreasing environmental contamination and recycling resources is a crucial challenge for humanity [1,5]. Citizens’ pro-environmental actions undoubtedly play a significant role in addressing this challenge [2,25]. In South Korea, the government is spearheading efforts to encourage eco-friendly consumption and recycling practices among citizens.

The South Korean government implemented the eco-labeling certification system in 1994 to promote the production and consumption of green products. Subsequently, in 2005, the “Act on the Promotion of Green Consumption” was enacted to stimulate citizens’ and public institutions’ interest in green products. This law mandates the purchase of products that have obtained environmental labeling or excellent recycling certification or that meet the corresponding criteria. As a result, the public procurement of green products steadily increased from USD 1.2 billion in 2008 to USD 3.2 billion in 2019. In addition, owing to citizens’ increased levels of environmental awareness, interest in green products has significantly increased. According to the 2016 and 2020 surveys on the environmental awareness of eco-friendly products conducted by the Korea Environment Institute [26,27], the general public’s interest in environmental issues rose from 53.9% in 2016 to 73.0% in 2020. The experience of purchasing green products also showed an upward trend, increasing from 15.5% in 2016 to 62.4% in 2020. Moreover, individual efforts to reduce the consumption of disposable products increased from 54.2% in 2016 to 78.4% in 2020.

The government’s “separate collection labeling” policy also encourages citizen participation in the sorting and collection of household refuse. This initiative classifies wastes based on recycling, incineration, and landfill disposal methods and labels them accordingly. Each type of waste is allotted a distinct color and symbol to facilitate the sorting and disposal process. For instance, plastic waste is labeled in blue, whereas glass waste is labeled in orange. These distinct collection labels facilitate proper classification and disposal by citizens at waste treatment facilities, encourage recycling, and contribute to the efficient use of resources. Consequently, South Korea’s municipal solid waste recycling rate remains the highest among the Organization for Economic Cooperation and Development (OECD) member nations. The most recent 2015 OECD municipal solid waste data indicate that the municipal solid waste recycling rates of the top three countries were 59% (South Korea), 66% (Germany), and 55% (Austria); thus, South Korea demonstrates an exceptional municipal solid waste recycling rate, even among the top-tier members [28].

## 3. Individualism and PEB

PEB refers to an individual’s purposeful actions to protect the environment [29]. It generally includes any environmentally friendly activities, both in the workplace and at home. For example, employees may voluntarily print on both sides of the page, turn off the lights, and use the stairs instead of elevators at work [2,30]. Individuals may also purchase energy-saving appliances, recycle household resources (e.g., glass bottles and batteries), and increase green consumption (e.g., buying organic food) in daily life [31]. Given that PEB is beneficial to natural environmental sustainability and community wellbeing [2,25], extensive research on environmental psychology has focused on the primary factors that motivate people to engage in PEB [1]. Indeed, many scholars have provided empirical evidence of various factors that affect PEB, such as political ideology [32], education [33], income level [34], and knowledge of environmental issues [35].

Despite the growing attention paid to promoting PEB, research on how differences in individual cultural values shape PEB remains scant [36]. Scholars distinguish cultural values into two sub-dimensions: personal levels of individualistic orientation and cultural levels of collectivistic orientation [37]. In this regard, Triandis ([38], p. 909) posited that “it should not be assumed that everybody in individualistic cultures has all the characteristics of these cultures, and that everyone in collectivistic cultures has the characteristics of those cultures”. Of particular interest to this study is individualism/collectivism among various personal values, as suggested by Hofstede [39]. Certain individual behaviors are visible physical reactions corresponding to the nature of psychological properties, including a person’s values, beliefs, and attitudes [6].

According to Hofstede [39], cultural values serve as fundamental factors in how people perceive themselves and others and how they interact with one another. Individualism and collectivism, in general, represent two opposite endpoints of a continuum [40]. In this respect, individualism (vs. collectivism) is the extent to which people are willing to behave as individuals (vs. members of a group or community) [41]. For individuals with high levels of individualism, for instance, their personal interests and goals are more important than those of the groups to which they belong [42]. Such individuals are motivated to conduct specific behaviors to enhance their own well-being and ego but are indifferent to in-group interests and responsibilities to their community [43]. By contrast, collectivists prioritize the interests of the group over personal interests, focus on the welfare of other members, and emphasize in-group harmony [44]. For people with collectivistic orientations, collective goals should be obtained even at the cost of individuals at large [6].

This opposing individualist–collectivist orientation plays a pivotal role in explaining PEB. VBN theory provides a useful framework for explaining the different effects of individualism and collectivism on PEB [45,46]. The theory assumes that individuals’ behavioral actions depend on underlying psychological concepts, such as their values and beliefs [47]. In particular, personal values are an initial guide for a person’s or society’s behaviors [48]. According to VBN theory, from which PEB originates, individual cultural values may influence the moral obligation to behave pro-environmentally for the welfare of other group members or the self-interest to satisfy individual desires [36]. For instance, collectivists seek to maximize collective interests, and they are susceptible to the impact of moral and social norms [49]. These features of collectivism allow people to make personal sacrifices for sustainability and donate cash or goods for environmental conservation purposes [6,36]. Similarly, individuals with a collectivistic orientation feel a sense of community belongingness and group loyalty that promotes behaviors beneficial to the environment, such as reducing waste and reusing goods, because collectivism is conceptually congruent with PEB [42]. Conversely, individualists are less likely to consider the influence of their behaviors on society when making decisions and thus have lower environmental awareness and attitudes than collectivists [49], which results in inaction to solve environmental problems. This means that individualists’ concentration on personal benefits and gains leads them to feel unconcerned about environmental protection in their daily lives or about the enhancement of their organization’s environmental performance at work [18]. On the basis of these arguments and previous empirical evidence, we propose the following hypothesis:

**Hypothesis** **1:** *Individualism is negatively associated with PEB*.

## 4. Moderating Role of Social Cohesion

Kawachi and Berkman ([50], p. 175) defined social cohesion as “the extent of connectedness and solidarity among groups in society.” In particular, the primary characteristic of social cohesion is that individuals have strong emotional ties of friendship, caring, liking, and proximity to their community (or groups) [51]. People in a socially cohesive community, for example, offer to help their neighbors and spend time together, and they thereby feel emotionally close to one another [52]. A community with high levels of social cohesion comprises residents who are driven to coordinate their efforts to attain their common goals for the sake of sustainable collective well-being [53]. That is, social cohesion allows individuals in groups or communities to actively participate in local voluntary associations that promote mutual communication and information sharing, which in turn form and strengthen trust in societal norms [54].

Social cohesion as a community asset may encourage individuals to show PEB. A strong network of ties between individuals encourages them to pay considerable attention to environmental protection issues through frequent interactions with neighbors in daily life and through participation in local voluntary associations [55,56]. In support of this rationale, Macias and Williams [57] revealed that people who spend social evenings with neighbors and relatives and who attend religious services are more likely to have environmentally friendly lifestyles, such as purchasing chemical-free produce, saving water and household energy, driving less, and avoiding non-green products. Social bonds expose collective individuals to similar sources of news and information in terms of environmental outcomes, and they share beliefs and values regarding environmental protection [58]. This means that a greater connection to others is associated with a greater willingness to perform PEB. Therefore, we formulated the following hypothesis:

**Hypothesis** **2:** *Social cohesion is positively associated with PEB*.

Social cohesion may not only affect PEB but also interact with individual cultural values to shape PEB. SET provides a basis for understanding how social cohesion moderates the relationship between individualism and PEB. According to Blau ([59], p. 91), social exchange refers to “voluntary actions of individuals that are motivated by the returns they are expected to bring and typically do in fact bring from others.” The idea of the theory is that exchange relationships characterized by the norm of reciprocity allow people to feel a sense of obligation to repay others who have helped or supported them [13,14]. Given that trust and helpfulness, which are key components of social cohesion, are the symbolic messages behind showing individualists goodwill gestures, social cohesion encourages individuals to reciprocate such favorable treatment from neighbors by exhibiting positive behaviors toward environmental sustainability protection and energy conservation [15,60,61]. Similarly, individualists feel that emotional connectedness for common welfare is beneficial for improving their personal well-being, which in turn motivates them to voluntarily behave in ways that improve the community’s environmental conditions [61,62]. The opposite may also be true. Individualists residing in a community with low levels of social cohesion are less likely to engage in PEB because their neighborhood environment, where people frequently experience distrust, isolation, or a lack of concern, makes them more likely to pursue personal gains rather than the common good [60]. Thus, we propose the following:

**Hypothesis** **3:** *Social cohesion moderates the negative relationship between individualism and PEB such that the relationship is weaker when the level of social cohesion is higher*.

## 5. Model Specification

### 5.1. Data Sources and Sample

To test our hypotheses, we employed the Korean General Social Survey (KGSS), which is identical to the U.S. General Social Survey of the National Opinion Research Center at the University of Chicago. The KGSS is regarded as a nationally representative survey with data based on the multi-stage area probability sampling of all Koreans aged 18 years and older. Of the series of KGSSs that have been collected since 2003, we relied on the 2021 survey because it provides the most recent data with items relevant to our study variables. The data were collected through face-to-face interviews, and the total response rate was 50%. After dropping cases with missing data for our study variables, we obtained a final sample of 1125. Table 1 presents the detailed background descriptions of the respondents.

### 5.2. Dependent Variable: PEB

According to Lange and Dewitte [3], PEB involves an individual’s acts that benefit the natural environment (e.g., recycling) and avoid harmful activities (e.g., the use of plastic). According to this definition, PEB in this study was measured using the following two items: (1) “*Make a special effort to sort glass, metals, plastic, paper, and so on for recycling*” and (2) “*Avoid buying certain products for environmental reasons.*” The score ranged from 1 to 5, with 1 representing the lowest level of effort toward PEB and 5 indicating the greatest level of effort. We averaged the individual responses to the two items.

### 5.3. Explanatory Variables

#### 5.3.1. Individualism

Individualism is defined as an individual’s tendency to be more concerned with personal goals and freedom, whereas collectivism refers to an individual’s tendency to be more concerned with the attainment of collective goals and to make self-sacrifices for public safety [63]. The level of individualism was manipulated through an item that captured individuals’ personal aims to follow coronavirus disease (COVID-19) guidelines, such as wearing a mask and observing social distancing: “*Between individual liberty and public safety, which COVID-19 guidelines should be prioritized more?*” The score ranged from 1 (personal freedom first) to 5 (public safety first). We reversely coded the responses to the item for analysis (i.e., 1 = public safety first and 5 = personal freedom first). Given that individualism and collectivism are at the opposite ends of a spectrum [64], respondents were considered individualists if their responses were close to 5 and collectivists if their responses were close to 1.

#### 5.3.2. Social Cohesion

As mentioned earlier, social cohesion refers to the degree of social connectedness among members in communities and societies and is characterized by mutual support and a sense of belonging to a neighborhood [50]. To measure social cohesion, we used the item “*We would like to ask about the area 1 km (approximately 15 min on foot) around your home. To what extent do you agree or disagree with each of the following statements?*” This was followed by two statements: (1) “*The neighbors are mutually concerned for each other*” and (2) “*The neighbors are willing to provide assistance when I am in need.*” Each item score ranged from 1 (always) to 5 (never). We reversely coded the responses to the two items for analysis (i.e., 1 = never and 5 = always). We averaged the individual responses to the two items.

### 5.4. Controls

We controlled for several predictors of PEB. First, we expected political ideology to affect individuals’ environmental sustainability behaviors [32]. Previous studies linking political ideology and PEB have reported that the political left is more likely to engage in sustainable consumption behaviors through political activities regarding ecological concerns than the political right [65]. Political ideology was measured using a single item: “*To what degree do you think of yourself as politically liberal or conservative?*” The score ranged from 1 (very liberal) to 5 (very conservative). Second, government trust, which might have a positive impact on PEB, was included in the models. If people believe that government institutions produce the desired outcomes, they will be more likely to perform environmentally friendly collective actions to support public policies for environmental protection [66]. We measured government trust using a single survey question from the KGGS: “*As far as the people running these institutions are concerned, would you say you have a great deal of confidence, only some confidence, or hardly any confidence at all in them?*” Respondents answered the two items, and the score for each ranged from 1 (hardly any confidence at all) to 3 (a great deal of confidence), which revealed their confidence in the following institutions: the executive branch of the national government and the local government. We averaged the individual responses to the two items. Third, household income levels were accounted for in our models. Individuals with higher levels of household income are more likely to perform PEBs because they have higher quality-of-life needs and a greater willingness to pay for environmental protection than those with low levels of household income [67]. We measured respondents’ household income levels using a single survey item: “*Compared to South Korean families in general, how far above or below the national average is your family income?*” The response categories ranged from 1 (much worse) to 5 (much better). Finally, the demographic characteristics of the respondents were included in our models because of their potential impact on PEB. These demographics were gender (1 = female, 0 = male), age (1 = less than 30 years, 2 = 30–39 years, 3 = 40–49 years, 4 = 50–59 years, 5 = 60+ years), and education (1 = high school, 2 = bachelor’s degree, 3 = graduate school).

### 5.5. Methodology

Before testing our hypotheses, we examined the descriptive statistics and Pearson correlation statistics among the variables used in the empirical analyses. Then, we used ordinary least squares (OLS) regression because our dependent variable comprised ratio scales, which were summed averages. Next, to better understand how the relationship between individualism and PEB changes depending on the level of social cohesion, we plotted the interaction effects between individualism and social cohesion by graphically comparing the plot lines obtained at one standard deviation below and above the mean [68]. Finally, we created marginal effect plots to ensure that the hypothesized interaction was thoroughly investigated [69].

## 6. Results

Table 2 provides the descriptive statistics (i.e., means and standard deviations), correlation coefficients, and results of the normality tests for all variables. Given that Pearson (parametric) correlation coefficients are sensitive to skewed distributions, the table also presents Spearman (non-parametric) correlation coefficients. The Spearman correlation coefficients are between variables above the diagonal, and the Pearson correlation coefficients are between variables below the diagonal. Pearson correlation is more suitable for testing the linear relationship between two continuous random variables, whereas Spearman’s rank-order correlation assesses the relationship between two (not necessarily continuous) random variables. Neither correlation coefficient differed significantly. As presented in the table, individualism was negatively correlated with PEB (*r* = −0.07, *p* < 0.05), whereas social cohesion was positively correlated with PEB (*r* = 0.18, *p* < 0.01). Gender (ρ = 0.12, *p* < 0.01), age (ρ = 0.12, *p* < 0.01), and household income levels (ρ = 0.07, *p* < 0.01) showed positive relationships with environmentally beneficial behaviors.

We also conducted Kolmogorov–Smirnov (K–S) tests to determine whether the variables were normally distributed. As all *p*-values were less than 0.05, the results indicated that the data did not follow a normal distribution. However, these tests often do not offer robust results regarding large-sampled data and variables with Likert-type scales [70,71]. For this reason, Groeneveld and Meeden [72] suggested assessing the skewness and kurtosis of the distribution as supplement methods for K–S tests. Although no cut-off values are agreed upon to determine whether a variable is normally distributed, building on skewness and kurtosis [73], scholars have recommended that if the absolute values of skewness are smaller than 2 or an absolute kurtosis is smaller than 7, the data can be evaluated to meet the normal distribution assumption [74]. Considering that our sample size was reasonably large and the main study variables were measured using Likert-type scales, we relied on the skewness and kurtosis values of the variables to determine the normality of the data. As shown in Table 2, the values of all variables were less than the cutoff values. Accordingly, we deemed that our data were distributed normally or close to normal.

Table 3 shows the results of the hierarchical linear regression analyses for PEB. Models 1 and 2 considered the linear terms of all variables, presenting the main effects of individualism and social cohesion on PEB. Model 3 added the interaction term individualism × social cohesion to inspect the moderating effects of social cohesion on the individualism–PEB link. Moreover, all our models computed robust standard errors using the Huber–White sandwich estimator to correct for heteroskedasticity and thereby obtain robust inferences regarding the estimated models.

With respect to the control variables, the results of Model 1 showed that gender was a significant predictor of PEB (β = 0.181, *p* < 0.01). Females were more willing to engage in environmentally friendly behaviors than males [35]. We also found that age (β = 0.102, *p* < 0.01) and education (β = 0.078, *p* < 0.05) were positively associated with PEB. Furthermore, the results provided evidence that household income had a positive impact on PEB (β = 0.087, *p* < 0.01).

In support of Hypotheses 1 and 2, Model 2 provided evidence that individualism was negatively associated with PEB (β = −0.056, *p* < 0.05), whereas social cohesion was positively associated with PEB (β = 0.108, *p* < 0.01). It is plausible that individuals with high levels of individualism are less likely to engage in PEB because they perceive ecological behaviors for improving environmental sustainability as unbeneficial to personal gain and self-interest [6,18]. Conversely, social cohesion encourages individuals to conduct PEB through mutual interactions with each other in terms of environmental protection and conservation because the improvement of environmental sustainability is a crucial collective goal [12].

We added the interactions between individualism and social cohesion in Model 3 to better explore SET. Our findings showed that social cohesion significantly moderated the negative association between individualism and PEB (β = 0.052, *p* < 0.05), which supports Hypothesis 3. It is possible that social cohesion leads individualists to reciprocate favorable treatment, such as care and support from neighbors, connectedness to others, and a sense of belongingness to the community, by performing environmentally friendly activities [15,60,61].

**Figure 1:**
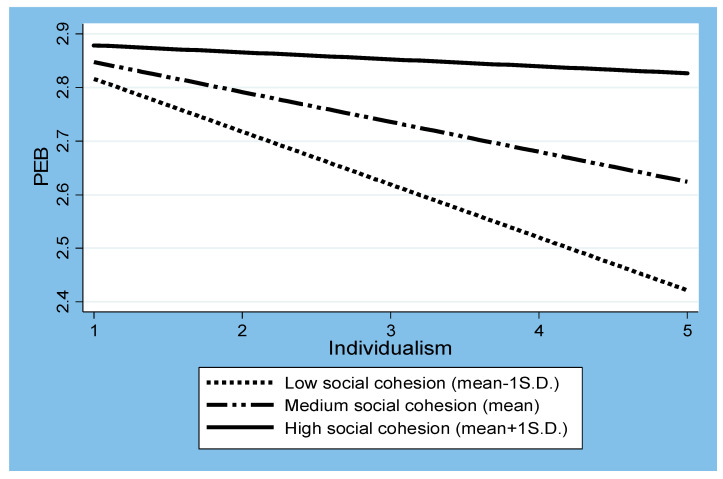
Interactions between individualism and social cohesion for PEB.

**Figure 2:**
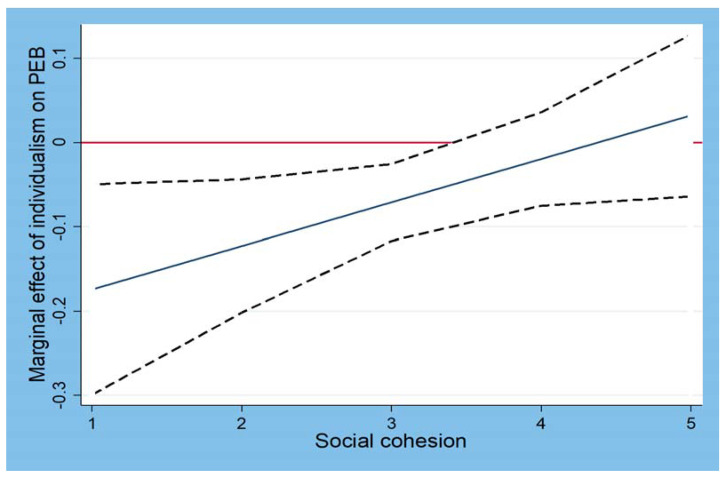
Interactions of individualism and social cohesion in predicting PEB.

We plotted how the interactions between individualism and social cohesion shape PEB, as shown in Figure 1. Consistent with Hypothesis 3, Figure 1 shows that when social cohesion was high—that is, one standard deviation (SD) above the mean (represented by the solid line)—the negative relationship between individualism and PEB was significantly weaker than when social cohesion was low—that is, one SD below the mean (represented by the dotted line). Similarly, Figure 2 visualizes in more detail how the relationship between individualism and PEB changed as social cohesion increased. The central line shows that the predicted PEB values depended on the varying levels of social cohesion when all the other variables included in our model were controlled for. The dotted lines indicate the upper and lower bounds of the 95% confidence intervals for the predicted PEB values. Accordingly, the area above the upper bound and below the horizontal zero line indicates the presence of a statistically significant relationship. The figure confirms that the negative relationship between individualism and PEB decreased as social cohesion increased. This negative effect of individualism on PEB became statistically indistinguishable from zero (where the upper confidence interval meets the zero line on the graph) when social cohesion was about one SD above the mean level (approximately 3.5).

## 7. Discussion

### 7.1. Main Findings and Implications

This study aimed to explore the effects of individualism and social cohesion on PEB and test social cohesion as a potential moderator in the relationship between individualism and PEB according to SET in the context of Korean society. The results showed that individualism had a negative relationship with PEB, whereas social cohesion had a positive relationship with PEB. Further analysis showed that social cohesion mitigated this negative relationship. These findings have not only important theoretical implications for individualism and PEB but also conclusive practical implications for promoting PEB to enhance environmental sustainability through building social cohesion.

First, some interesting findings emerged with respect to the control variables. Gender was positively associated with PEB. According to gender socialization theory, women are socialized to be nurturing, empathetic, and cooperative, whereas men are taught to be competitive, aggressive, and emotionally distant from others [75]. Furthermore, according to the findings of previous studies, women are perceived to be more collectivist than men. That is, women exhibit a greater inclination and ability to provide care to others, and they define their identities on the basis of their connections with others, indicating a tendency toward collectivism. This woman–nature association leads to women engaging more in PEB than men [76]. The results also showed that age and education were positively related to PEB. It may be plausible that highly educated people are more likely to conduct PEB than those who have low levels of education because education increases people’s environmental awareness and knowledge [77]. In terms of age, older people were more likely to perform PEB than younger ones because people become more environmentally oriented as they get older [78]. Household income level was significantly and positively associated with PEB. This may be interpreted to mean that people with high levels of income have their basic needs (e.g., housing and medical care) satisfied and can thus seek to improve environmental quality as a way of increasing their quality of life [79].

Second, this study demonstrates that personal values drive PEB. Extending previous studies on PEB, the current research advances our understanding of the underlying mechanism through which personal values could shape PEB. VBN theory suggests that individuals’ behaviors are contingent on underlying psychological determinants, such as values, beliefs, and intentions [47]. According to Hofstede [39], individualism is characterized by a preference for weak connectedness in which individuals take care of their self-interests, whereas collectivism emphasizes strong connectedness in which individuals are willing to achieve a common interest. This implies that PEB may be strongly influenced by the values individuals pursue—individualism or collectivism. In this respect, our evidence of the negative relationship between individualism and PEB indicates that individualists are less likely to engage in PEB because they are independent of one another and focused on personal gains, which is consistent with the tenet of VBN theory [45,46]. Accordingly, our study suggests that VBN theory is effective for understanding how personal values shape PEB.

Third, our research advances the existing literature on the predictors of PEB by exploring the effect of social cohesion on PEB as well as the moderating role of social cohesion on the relationship between individualism and PEB according to the main idea of SET. Thus, we enrich theoretical perspectives to explain the individualism–PEB link based on social cohesion. Indeed, our findings are encouraging and theoretically consistent. In particular, the findings show that social cohesion not only has a positive impact on PEB but also moderates the negative association between individualism and PEB. We recognize that social cohesion, as a key collective asset of the community, promotes individuals’ PEB [12,17]. That is, social processes characterized by supportive resident interactions—being caring and willing to help each other—lead to individuals having greater concerns and awareness of the environment because attention to environmental sustainability is congruent with community wellbeing [10]. This means that more supportive social interactions lead to a greater willingness to engage in environmentally friendly behaviors [58,60]. Furthermore, our study contributes to extant research by proposing the essential role of social cohesion in attenuating the negative association between individualism and PEB. Building on the norm of reciprocity in SET, social cohesion results in individuals feeling obligated to repay favorable treatment, such as care and assistance received from neighbors, by showing behaviors beneficial to the improvement of the community environment [15,60,61]. Although individualists are less likely to perform PEB because of their pursuit of personal gains, they are willing to pay attention to environmental conservation issues and engage in eco-friendly behaviors in daily life when they experience favorable treatment in their community [61,62]. This implies that social cohesion plays a pivotal role in inducing individualists to conduct PEB by internalizing a sense of reciprocity.

Finally, our findings also suggest that a community needs to be a living collective [12,80] and that community cohesion is a critical component in promoting community members’ PEB. We know that social cohesion facilitates individualists’ PEB. Therefore, policymakers and community organizers should implement various community-based programs to improve social cohesion levels. For example, given that participation in local decision-making processes strengthens a sense of belonging and togetherness among inhabitants, community organizers need to hold various types of community meetings, such as town hall meetings and forums. Furthermore, community-based events, such as music festivals and exhibitions, may enhance social connections and encourage individualists to expand their social networks and develop trust in their neighborhoods. We recommend community-based physical activities, such as biking, walking, and jogging, to be organized as important avenues for increasing social cohesion and thus mitigating the negative effects of individualism on PEB.

### 7.2. Study Limitations and Suggestions for Future Research

Several limitations of this study should be addressed. First, we acknowledge that the measure of individualism was not constructed through the widely used multiple items developed by Yoo et al. [81] to capture the characteristics of individualism, in which individuals put more emphasis on their personal goals and welfare than on those of their community. Social cohesion was also not measured using multiple items asking whether the people in the neighborhood trust one another, whether respondents have neighbors who are capable of providing assistance during emergency situations, or whether respondents think that the people in the neighborhood care for one another [82]. We also recognize that the measurement of PEB relied on only two items that reflected behavioral efforts in recycling reusable waste products and minimizing the use of environmentally unfriendly products, which means that our dependent variable could not adequately measure the multiple dimensions of PEB. Therefore, our measures could be criticized in terms of their construct validity. This measurement issue is often encountered when secondary data, such as KGSS, are used to investigate the general perceptions of citizens on society but do not include the specific components of cultural values and tightly knit interpersonal relationships in the community. Hence, future research needs to improve the measures of individualism, social cohesion, and PEB by employing multiple items that precisely reflect their conceptual features.

Second, although the correlation between PEB and individualism was not strong, autocorrelation may exist between the two variables because the individualism measurement in this study, which captures the extent to which an individual is willing to follow COVID-19 guidelines for the sake of one’s own safety or public safety, could share common attitudinal features with PEB. That is, people who had a high level of individualistic orientation in the previous year may be less likely to engage in PEB in the current year.

Third, we relied on individualism at the individual level, but this personal value can be shaped by the norms and culture of the society at the collective level [83]. Consequently, future research should use different longitudinal data sources to measure the study variables, thus avoiding potential autocorrelation issues, and employ multilevel regression models that are beneficial to testing hypothesized relationships between variables measured at different levels (e.g., individualism at the individual level and cultural values at the collective level).

Finally, our results showed weak coefficients of determination (R-squared), which indicates the presence of other determinants with considerable influence on PEB. For example, previous studies have provided empirical evidence that environmental attitudes (e.g., perceptions of climate change and environmental protection) [84], personality (e.g., openness to experience and agreeableness) [85], and government policies (e.g., environmental regulations and provision of environmental information) [86] are significant antecedents of PEB. Many other potential moderators, including gender [87], social media exposure [88], and environmental knowledge [89], could also moderate the relationships between the antecedents and PEB. Thus, future researchers need to consider other factors that may have strong effects on PEB and thus offer more meaningful theoretical and practical implications for stakeholders.

## 8. Conclusions

The topic of PEB has garnered increased public attention owing to the swiftly deteriorating natural environment, and the threats caused by global warming, biodiversity loss, air pollution, and so on are more severe than ever [1,90]. This means that PEB could not only improve our psychological well-being but also promote environmental sustainability [91]. Hence, exploring the factors and underlying mechanisms that shape individuals’ PEB is important [92]. Indeed, many studies have examined various antecedents of individuals’ behavior that benefit the environment, but little is known about how individual psychological factors shape PEB. Given that personal values are more predictive of PEB than external factors (such as economic costs and convenience) and demographic variables [1], we paid special attention to the impact of individualism on PEB. We also investigated the contingent effect of social cohesion on the relationship between individualism and PEB because individuals’ behaviors can be conceived as a product of the interactions between personal values and subjective perceptions of the social environment (i.e., social cohesion) [51]. By addressing these research gaps, this study made a significant contribution to advancing our knowledge of how individualism influences PEB and how social cohesion conditions the individualism–PEB link. Regarding public policy, we suggest that policymakers tailor their community policies and services to facilitate emotional care and helping behaviors among residents.

## Figures and Tables

**Table 1:** Background descriptions of the respondents.

Background	Category	n	%
Gender	Female	650	57.78
	Male	475	42.22
Age	<30	115	10.22
	30–39	147	13.07
	40–49	180	16.00
	50–59	281	24.98
	≥60	402	35.73
Education	High school or less	668	59.38
	Bachelor’s degree	432	38.40
	Graduate degree	25	2.22
Household income	Very satisfied	3	0.27
	Somewhat satisfied	120	10.67
	Neither satisfied nor dissatisfied	559	49.69
	Somewhat dissatisfied	372	33.07
	Very dissatisfied	71	6.31
Political ideology	Very liberal	61	5.42
	Somewhat liberal	309	27.47
	Neither liberal nor conservative	432	38.40
	Somewhat conservative	261	23.20
	Very conservative	62	5.51
	Total	1125	100

**Table 2:** Descriptive statistics, correlations, and normality tests.

	Variable	(1)	(2)	(3)	(4)	(5)	(6)	(7)	(8)	(9)
(1)	Pro-environmental behavior	1.00	−0.06	0.18	−0.01 ^a^	−0.01 ^a^	0.12	0.12	−0.02 ^a^	0.07
(2)	Individualism	−0.07	1.00	0.00	0.04 ^a^	−0.03 ^a^	−0.03 ^a^	0.03 ^a^	0.01 ^a^	−0.05 ^a^
(3)	Social cohesion	0.18	−0.01 ^a^	1.00	0.06	0.03 ^a^	0.10	0.22	−0.12	0.04 ^a^
(4)	Government trust	−0.01 ^a^	−0.04 ^a^	0.08	1.00	−0.08	0.04 ^a^	0.04 ^a^	−0.05 ^a^	0.02 ^a^
(5)	Political ideology	0.00 ^a^	0.03 ^a^	0.04 ^a^	−0.09	1.00	−0.02 ^a^	0.27	−0.14	−0.07
(6)	Gender (Female = 1)	0.13	0.03 ^a^	0.10	0.04 ^a^	−0.01 ^a^	1.00	0.00 ^a^	−0.11	−0.01 ^a^
(7)	Age	0.15	−0.02 ^a^	0.22	0.05	0.25	0.03 ^a^	1.00	−0.51	−0.22
(8)	Education	−0.15 ^a^	−0.01 ^a^	−0.11	0.07	−0.13	−0.10	−0.48	1.00	0.28
(9)	Household income	0.08	0.04 ^a^	0.05	0.02 ^a^	−0.06	−0.01 ^a^	−0.20	0.29	1.00
	Mean	2.70	2.40	3.30	5.97	2.95	0.58	3.63	1.42	2.65
	SD	0.88	0.88	0.83	1.60	0.97	0.49	1.35	0.54	0.77
	Skewness	0.04	−0.45	−0.24	0.18	0.09	−0.31	−0.62	0.71	−0.15
	Kurtosis	2.73	2.15	2.67	2.58	2.54	1.10	2.14	2.35	2.87
	K–S	0.18	0.33	0.16	0.26	0.20	0.38	0.22	0.38	0.28

Note: ^a^ Not significant at a 95% confidence level; SD = standard deviation; K–S = Kolmogorov–Smirnov statistics.

**Table 3:** OLS regression results for the hypothesized relationships.

	Model 1			Model 2			Model 3		
	β		S.E.	β		S.E.	β		S.E.
Gender (Female = 1)	0.181	***	0.039	0.166	***	0.039	0.170	***	0.039
Age	0.102	***	0.017	0.086	***	0.170	0.086	***	0.017
Education	0.078	**	0.043	0.077	**	0.042	0.074	**	0.042
Household income	0.087	***	0.027	0.081	***	0.027	0.079	***	0.027
Political ideology	−0.024		0.021	−0.022		0.021	−0.023		0.021
Government trust	−0.035		0.036	−0.049		0.036	−0.053		0.036
Social cohesion				0.108	***	0.024	−0.014		0.066
Individualism				−0.056	**	0.022	−0.226	**	0.088
Individualism x social cohesion							0.052	**	0.026
Constant	2.082	***	0.149	1.964	***	0.222	2.382	***	0.268
R^2^	0.054			0.070			0.080		
N	1125			1125			1125		

Note: ** *p* < 0.05; *** *p* < 0.01.

## Data Availability

The data used for this study are available at https://kgss.skku.edu/kgss/data.do, accessed on 15 April 2023).
